# Hemorrhagic Pericardial Effusion and Gastrointestinal Bleed After Atrial Fibrillation Epicardial Ablation: A Case Report

**DOI:** 10.1002/ccr3.71501

**Published:** 2025-11-19

**Authors:** Kairui Sun

**Affiliations:** ^1^ Michigan State University College of Osteopathic Medicine USA

**Keywords:** atrial fibrillation, case report, epicardial ablation, gastrointestinal bleeding, hemorrhagic pericardial effusion

## Abstract

A 47‐year‐old male with persistent atrial fibrillation presented with melena and presyncope 3 weeks after epicardial ablation and left atrial appendage ligation. He was found to have hemorrhagic pericardial effusion with tamponade physiology and gastrointestinal bleeding from a clean‐based duodenal ulcer. Management included pericardiocentesis, anticoagulation reversal, and stabilization. This case underscores the interplay between cardiac tamponade and GI bleeding, highlighting the importance of early recognition and multidisciplinary management.

**Summary**

- Delayed hemorrhagic pericardial effusion after epicardial AF ablation may precipitate stress‐related duodenal ulceration.
- Early tamponade detection, prompt anticoagulation reversal, and coordinated multidisciplinary care are critical to mitigate concurrent cardiac and gastrointestinal complications.

## 1 Introduction

Atrial fibrillation (AF) is a common arrhythmia associated with significant morbidity. Hybrid ablation combining endocardial and epicardial approaches is increasingly utilized for refractory cases. While effective for treating certain cardiac arrhythmias, it is associated with several potential complications. Pericardial bleeding is a common complication, with rates reported around 5% in some studies. This can result from unintentional cardiac puncture or laceration of coronary vessels. Cardiac tamponade, a severe form of pericardial bleeding, may require emergent surgical intervention [1, 2, 3]. Coronary artery injury can occur during catheter manipulation, leading to coronary spasm or even occlusion. This complication, although less frequent, can result in myocardial infarction if not promptly managed [1, 4]. Other complications include phrenic nerve injury, which can cause diaphragmatic paralysis, and hepatic injury, such as subcapsular hematoma or liver laceration, due to the anatomical proximity of the liver to the pericardial space. Additionally, pleural perforation and hemoperitoneum have been reported, particularly with inferior‐oriented puncture techniques [4, 5, 6, 7]. Delayed complications include pericarditis and pericardial adhesions, which can complicate future procedures and increase the risk of recurrence of arrhythmias [3, 6, 7].

The literature indicates that gastrointestinal complications, including bleeding, can occur as a result of epicardial ablation procedures. For instance, Koruth et al. (2011) describe intra‐abdominal bleeding related to puncture of the liver during epicardial access, which required surgical repair [4]. Additionally, Killu et al. (2013) report hemoperitoneum and abdominal–pericardial fistula as complications, which could potentially lead to gastrointestinal bleeding [6]. However, to our knowledge, there is no report of GI ulcer and bleed in the setting of pericardial effusion as a delayed complication from epicardial ablation.

**FIGURE 1:**
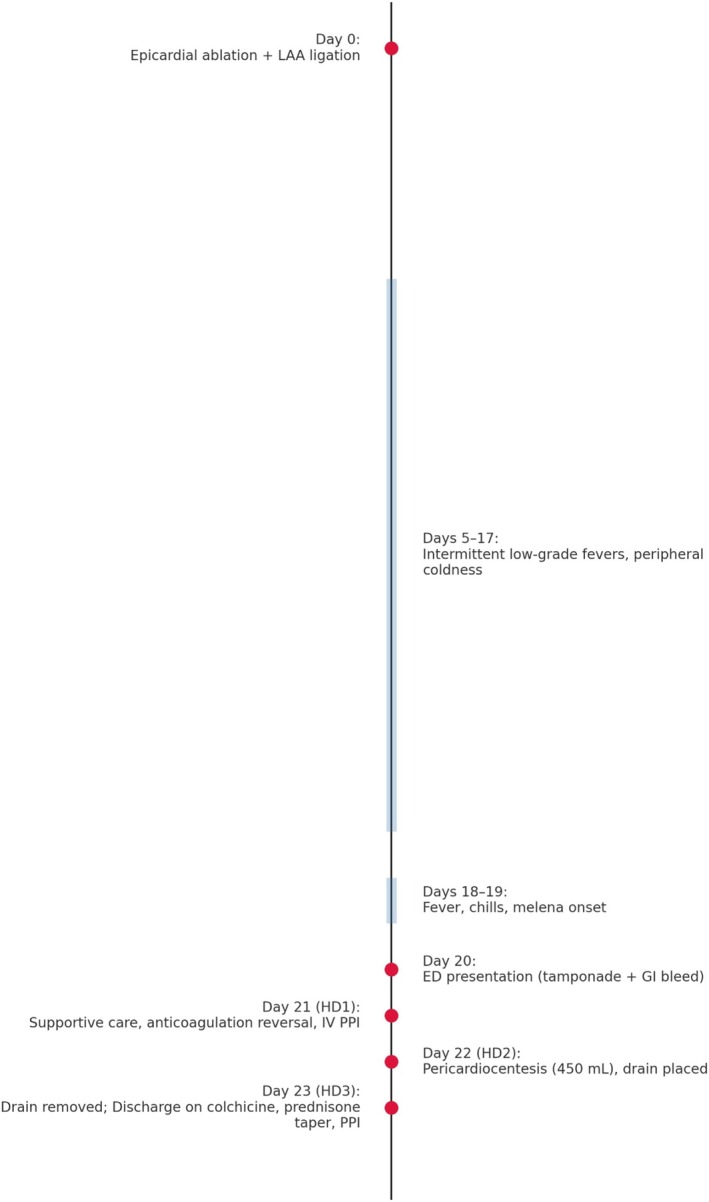
Clinical timeline summarizing presentation, interventions, and outcomes.

Here, we present a case of hemorrhagic pericardial effusion and GI bleeding following epicardial ablation (Figure 1).

## 2 Case Presentation

### 2.1 Patient History

A 47‐year‐old male was diagnosed with atrial fibrillation in his 30s. Comorbidities included hypertension and hyperlipidemia; there was no history of peptic ulcer disease, chronic NSAID use, corticosteroid use prior to the procedure, or alcohol abuse. He experienced palpitations and fatigue during episodes and underwent approximately 10 cardioversions over a decade. Several years ago, he underwent an endocardial ablation, achieving nearly 2 years of sinus rhythm. However, symptomatic AF recurred, and he reported intolerance to medical therapy due to side effects. Epicardial access was pursued due to prior endocardial ablation failure, persistent symptomatic AF, and an enlarged left atrium. This approach is supported in refractory AF by Heart Rhythm Society and European Heart Rhythm Association consensus statements [1, 2]. Before the procedure, a stress test was negative, and a transthoracic echocardiogram (TTE) showed a normal left ventricular ejection fraction (LVEF) and no valvular abnormalities. Several weeks prior to presentation, he underwent a planned hybrid ablation with left atrial appendage ligation. Postoperatively, he maintained normal sinus rhythm (NSR) without immediate complications. To minimize the risk of postoperative inflammation, he was initiated on colchicine and methylprednisolone following the convergent procedure.

### 2.2 Hospital Course

In the weeks following the procedure, the patient experienced intermittent low‐grade fevers up to 37.9°C (100.3°F), and peripheral coldness. Approximately 3 weeks post‐procedure, he developed fever, chills, fatigue, weakness, presyncope, and a large melena bowel movement, prompting hospital admission.

On ED arrival, BP was 92/60 mmHg, HR 108 bpm, temp 38.5°C (101.3°F), RR 20/min, SpO_2_ 97% on room air. He was pale and diaphoretic but without abdominal tenderness. He denied abdominal pain, nausea, vomiting, chest pain, shortness of breath, or dysuria. A summarized timeline of presentation and management is provided (Table 1).

**TABLE 1:** Timeline of Patient's Presentation.

Date	Event
Day 0	Epicardial ablation with left atrial appendage ligation; started on colchicine and methylprednisolone.
Day 5–17	Intermittent low‐grade fevers and peripheral coldness.
Day 18–19	Fever, chills, fatigue, weakness, presyncope, and onset of melena.
Day 20	Presentation to ED with fever, hypotension, and tachycardia; large pericardial effusion identified on TTE.
Hospital Day 1	Admission to hospital; initiation of supportive care, anticoagulation reversal, and PPI therapy.
Hospital Day 2	Pericardiocentesis performed; 450 mL hemorrhagic fluid drained; pericardial drain placed.
Hospital Day 3	Pericardial drain removed following stabilization.
Hospital Discharge (~Day 23)	Discharged on colchicine, prednisone taper, and oral PPI; follow‐up planned for anticoagulation resumption.

### 2.3 Investigations

- Hemoglobin: 7.5 g/dL (baseline 14 g/dL)
- Transthoracic echocardiogram: large lobulated pericardial effusion with right atrial and ventricular diastolic collapse (tamponade physiology)
- EGD: clean‐based duodenal ulcer (Forrest III), no active bleeding, no visible vessel; gastric and duodenal mucosa otherwise normal. No stigmata of portal hypertension.

Pericardiocentesis was performed on the second day of admission, draining 450 mL of bloody fluid, with a pericardial drain placed and subsequently removed on admission day three following stabilization. Pericardial fluid cytology and cultures were negative; fluid was not grossly purulent. Anticoagulation with rivaroxaban was reversed using four‐factor prothrombin complex concentrate. The patient received 2 units of packed red blood cells with symptomatic improvement. Proton pump inhibitor (PPI) therapy with IV pantoprazole was initiated. Features of pericarditis were noted and managed with colchicine and a prednisone taper. The patient stabilized hemodynamically, was discharged on colchicine, prednisone taper, and oral PPI, with instructions for follow‐up echocardiography, CBC (complete blood count) to monitor for recurrent anemia, and reevaluation for anticoagulation resumption in 6 weeks per AF/pericardiocentesis guideline recommendations [8, 9].

## 3 Discussion

This case demonstrates a rare presentation of GI bleeding temporally associated with delayed hemorrhagic pericardial effusion following epicardial AF ablation. While causality cannot be definitively established, tamponade‐related hemodynamic compromise likely contributed to mucosal ischemia and stress‐related ulceration, particularly in the duodenum's watershed zones.

Hemodynamic instability from hemorrhagic pericardial effusion likely reduced cardiac output, creating a physiology analogous to cardiogenic shock. This can precipitate splanchnic hypoperfusion through renin–angiotensin–mediated vasoconstriction, restricting gastric and duodenal blood flow. In a porcine model, Bailey et al. demonstrated that such vasospasm disproportionately compromised gastric perfusion, leading to mucosal necrosis, hemorrhage, and ulceration; inhibition of the renin–angiotensin axis reduced these injuries, underscoring its role in stress ulceration [10]. More broadly, stress ulcer pathophysiology in cardiogenic shock is multifactorial: systemic hypoperfusion and compensatory vasoconstriction cause mucosal ischemia and barrier breakdown, while microcirculatory dysfunction perpetuates hypoxia even after partial hemodynamic recovery. Inflammatory activation and cytokine release further impair endothelial integrity and protective defenses such as bicarbonate and mucus secretion, increasing vulnerability to acid‐mediated injury. The combined effects of ischemia, microvascular dysfunction, and inflammation ultimately promote stress‐related mucosal damage and ulceration [10, 11, 12, 13, 14].

Anatomically, there is no direct vascular structure between epicardial puncture sites and the duodenum that would explain an immediate mechanical cause for ulcer formation; thus, an indirect hemodynamic mechanism is more plausible. Anticoagulation further increases bleeding risk.

### 3.1 Pericarditis Prophylaxis and Corticosteroid Considerations After Epicardial Ablation

Guidelines support colchicine as the preferred agent for pericarditis prophylaxis after epicardial ablation, while corticosteroids are not recommended for routine use. The 2017 Heart Rhythm Society consensus statement identifies colchicine as the cornerstone of pericarditis management and cites randomized trial data supporting a 3‐month course (0.5 mg twice daily) for reducing recurrence and improving quality of life after AF ablation, with dose adjustments for patients < 70 kg or with renal impairment [14]. Corticosteroids should be reserved for patients who fail or cannot tolerate NSAIDs and colchicine, as endorsed by the ACC/AHA, and when used, low‐to‐moderate doses (prednisone 0.2–0.5 mg/kg/day) are preferred due to lower recurrence and adverse event rates compared to high‐dose regimens [15, 16]. Importantly, systemic corticosteroids such as methylprednisolone can increase the risk of stress ulcers and gastrointestinal bleeding, particularly in high‐risk patients with cardiogenic shock, mechanical ventilation, or concurrent anticoagulation [17, 18, 19]. Observational and randomized studies in cardiac surgery and ablation settings have shown variable but concerning rates of GI bleeding with corticosteroid use, and guideline recommendations emphasize limiting dose and duration while providing stress ulcer prophylaxis (e.g., proton pump inhibitor) in high‐risk cases [17, 18, 19].

### 3.2 Anticoagulation Resumption

There is no fixed guideline‐mandated interval for restarting anticoagulation after pericardiocentesis in atrial fibrillation, particularly when stress ulcer risk and cardiogenic shock are present. Current recommendations emphasize resuming only after complete hemostasis and when bleeding risk is acceptably low, balancing this against thromboembolic risk [8, 9]. For high‐risk patients (e.g., CHA_2_DS_2_‐VASc ≥ 4), the ACC/AHA advise early resumption once safe, but not before hemostasis is confirmed [8, 9, 20]. After gastrointestinal bleeding, observational data suggest resumption within 1–3 weeks may balance stroke prevention and rebleeding risk, though timing should be individualized and guided by multidisciplinary input [21].

## 4 Conclusion

Epicardial ablation for refractory atrial fibrillation can be associated with serious complications, including hemorrhagic pericardial effusion and gastrointestinal bleeding. In this case, tamponade‐related hemodynamic compromise, compounded by corticosteroid therapy for post‐procedural pericarditis prophylaxis, may have contributed to stress‐related duodenal ulceration. While this case suggests a potential association, definitive causality cannot be established. Clinicians should remain vigilant for such presentations. Early recognition, supportive care, and a multidisciplinary approach are critical for optimizing outcomes.

## Author Contributions

**Kairui Sun:** conceptualization, data curation, formal analysis, investigation, methodology, writing – original draft.

## Consent

Written informed consent was obtained from the patient for the publication of this case report, including any clinical details. The patient was assured that all identifying information would be omitted to ensure their confidentiality and privacy.

## Conflicts of Interest

The authors declare no conflicts of interest.

## Data Availability

The data that support the findings of this study are available from the corresponding author upon reasonable request. The data are not publicly available due to privacy and ethical restrictions.
